# n-3 PUFA ameliorate functional outcomes following repetitive mTBI in the *fat-1* mouse model

**DOI:** 10.3389/fnut.2024.1410884

**Published:** 2024-07-12

**Authors:** Jessi S. Lau, Cody A. C. Lust, Jessica-Dominique Lecques, Lyn M. Hillyer, Margo Mountjoy, Jing X. Kang, Lindsay E. Robinson, David W. L. Ma

**Affiliations:** ^1^Department of Human Health and Nutritional Sciences, University of Guelph, Guelph, ON, Canada; ^2^Department of Family Medicine, McMaster University, Hamilton, ON, Canada; ^3^Massachusetts General Hospital and Harvard Medical School, Boston, MA, United States; ^4^Omega-3 and Global Health Institute, Boston, MA, United States

**Keywords:** repetitive, mild traumatic brain injury, omega 3 (n-3) polyunsaturated fatty acids, concussion, inflammation

## Abstract

**Purpose:**

Repeated mild traumatic brain injuries (mTBI) are a continuing healthcare concern worldwide, given its potential for enduring adverse neurodegenerative conditions. Past research suggests a potential protective effect of n-3 polyunsaturated fatty acids (PUFA) in experimental models of mTBI. The aim of this study was to investigate whether the neuroprotective benefits of n-3 PUFA persist following repetitive weight drop injury (WDI).

**Methods:**

Male *fat-1* mice (*n* = 12), able to endogenously convert n-6 PUFA to n-3 PUFA, and their wild type (WT) counterparts (*n* = 12) were maintained on a 10% w/w safflower diet. At 9–10 weeks of age, both groups received one mild low-impact WDI on the closed cranium daily, for three consecutive days. Following each WDI, time to righting reflex and seeking behaviour were measured. Neurological recovery, cognitive, motor, and neurobehavioural outcomes were assessed using the Neurological Severity Score (NSS) over 7 days (168 h) post-last WDI. Brains were assessed for cerebral microhemorrhages by Prussian blue and cellular damage by glial fibrillary acidic protein (GFAP) staining.

**Results:**

*Fat-1* mice exhibited significantly faster righting reflex and seeking behaviour time, and lower mean NSS scores and at all post-WDI time points (*p* ≤ 0.05) compared to WT mice. Immunohistochemistry showed no significant difference in presence of cerebral microhemorrhage however, *fat-1* mice had significantly lower GFAP staining in comparison to WT mice (*p* ≤ 0.05).

**Conclusion:**

n-3 PUFA is effective in restoring cognitive, motor, and behavioural function after repetitive WDI, which may be mediated through reduced cellular damage of the brain.

## 1 Introduction

Mild traumatic brain injury (mTBI) stands out as a prevalent neurological disorder and as the predominant form of traumatic brain injury (TBI), consisting of 70–90% of brain injuries (1). While frequently used interchangeably, concussions are classified as a subgroup within mTBI; nevertheless, it is important to note that mTBI does not always manifest as a concussion (2, 3). The defining features of mTBI involve a non-life-threatening impact, either direct or indirect, to the head, lacking any indication of skull fracture, alongside a Glasgow Coma Scale score ranging from 13 to 15 (2, 4, 5). The manifestations of mTBI encompass a diverse array of symptoms, spanning in severity from headaches, sleep disturbances, challenges in memory retention, to persistent cognitive impairments (6). While the symptoms of acute mTBI often subside after several months, the increased likelihood of developing permanent sequelae such as long-term cognitive and neurobehavioural impairments, is observed with repetitive mild insults (6, 7). Thus, given the potential cumulative effects of such exposures, there is rising concern about the long-term adverse consequences associated with repeated mTBI and to sub-concussive impacts, where no apparent signs or symptoms are evident following a head impact (7, 8).

Across Canada and the United States, epidemiological studies report an annual incidence of mTBI ranging between 200,000 and 1.6–3.8 million, respectively (9, 10). However, in reality, estimates of incidence are projected to be much higher due to underreporting and lack of acknowledgement by individuals regarding the severity of the injury (11). Consequently, it is important to understand both the acute response profile following an mTBI and long-term risk in affected individuals. Until recently, a significant portion of research directed towards mitigating the pathophysiological response to injury has concentrated on pharmaceutical interventions post-injury. However, there is no substantiated evidence supporting the efficacy of pharmacological therapy in reducing recovery duration or offering neuroprotection before the occurrence of an impact (12, 13). Acknowledging the inherent constraints in existing treatment methods and recognizing the necessity for proactive preventive measures, research efforts have expanded towards strategies involving nutraceuticals and nutritional supplementation (14).

Previous studies indicate that n-3 polyunsaturated fatty acids (n-3 PUFA), such as eicosapentaenoic acid (EPA) and docosahexaenoic acid (DHA) and, might prove beneficial in protecting the brain against mTBI (15). The essential and major dietary form of n-3 PUFA is alpha-linolenic acid (ALA) (16). Although EPA and DHA are downstream products metabolized from ALA (17), conversion rates are said to be below 1%, with EPA conversion being higher than DHA (18, 19). Thus, obtaining EPA and DHA through marine-based dietary sources is more efficient (16). DHA composes over 90% of the n-3 PUFA content and approximately 10–20% of the total lipids in the human brain (19, 20). As a result, DHA plays a vital role as a structural component in plasma membranes, contributes to neuronal signaling, and shares anti-inflammatory properties with EPA (17).

Prior research conducted by our lab demonstrated in the *fat-1* mouse model, which is capable of *de novo* synthesis of n-3 PUFA from n-6 PUFA (21), has shown improvements in functional outcomes after a single mild weight drop injury (WDI) (15). This initial study was a proof of concept study to establish a model that could later be used to further study the effects of n-3 PUFA and conditions such as multiple repeated mTBI observed in football, rugby, and hockey. Therefore, in the present study, we aimed to ascertain whether the effects of n-3 PUFA persist in the context of repetitive mTBI using the novel *fat-1* transgenic mouse model. The *fat-1* mouse is capable of synthesizing n-3 from a single diet enriched in n-6 PUFA. Building upon the findings observed in our previous study involving a single WDI, wherein significant neurological recovery was evident in *fat-1* mice, we hypothesized that *fat-1* mice will continue to display better outcomes after three consecutive WDI.

## 2 Materials and methods

### 2.1 Animals, housing, and experimental design

All procedures related to the care, handling, and experimentation of animals were carried out in accordance with and approval from the Animal Care and Use Committee of the University of Guelph (AUP #4207). Male C57BL/6 *fat-1* mice were bred with C57/Bl6 female mice, acquired from Charles River, to produce *fat-1* and WT progeny. Post-weaning at 3 weeks, offspring were fed a diet consisting of 10% w/w safflower (D04092701, Research Diets, New Brunswick, NJ, United States) and given distilled water *ad libitum*. Housing conditions included group accommodations of 3–4 mice per cage. The environment was regulated to maintain a temperature range of 22–24°C, and 12 h light/dark cycle, with experimental testing occurring exclusively in the light periods. When mice reached 9 to 10 weeks of age, male *fat-1* and WT mice (*n* = 24), were exposed to one WDI, for three consecutive days. After the third WDI, mice were monitored for a span of 1 week (i.e., 168 h), during which their motor, cognitive, and neurobehavioural functions were evaluated before being euthanized for tissue collection. WT control and *fat-1* control (*n* = 24), underwent all experimental procedures with the exclusion of the repetitive WDI.

### 2.2 Inducing three consecutive mild traumatic brain injury

Mice were exposed to a singular WDI for three consecutive days as established by Flierl et al. (22). Anesthesia induction involved the administration of 4% Baxter isoflurane (Cat #19476, CMDV, St. Hyacinthe, Quebec) vaporized in oxygen. Upon achieving the surgical plane (between 45 and 105 s), mice were removed from the anesthetic chamber and placed within a nose cone. The isoflurane concentration was reduced to a maintenance level of 1.5% until pedal reflexes ceased, totaling a maximum of 4 min under anesthesia. Anesthesia was terminated just before initiating the WDI. The induction of a single concussive impact included placing the anesthetized mouse chest-down on a slit-piece of aluminum foil, positioned 10 cm above a foam cushion and secured by a Plexiglass apparatus. The alignment ensured that a 100 g steel weight (1.3 cm × 28 cm) fell directly onto the dorsal surface of the scalp, midline between the bregma and lambda. Concussion administration involved rapidly elevating the weight using a nylon fly fishing line. Subsequently, the weight was released vertically from a height of 163 cm through a PVC guide tube (20 mm × 163 cm), delivering a force of impact clinically relevant to closed cranium scenarios (15). This single WDI design was repeated at the same time on the following 2 days, resulting in a total of three mTBI on successive days.

### 2.3 Time to first movement – righting reflex and seeking behaviour

Following all three WDI, each mouse was promptly relocated to a containment enclosure and positioned on their dorsal side. Subsequently, the righting reflex duration was assessed, measuring the time required for the mouse to reorient itself, transitioning from dorsal to ventral positioning. A secondary time was recorded to gauge the seeking behaviour of the mouse. The time to first movements were evaluated as an indicator of neurological recovery, akin to the righting reflex parameter assessed in the study by Kane et al. (23).

### 2.4 Neurological severity score

Mice were evaluated for both functional recovery and the extent of mTBI induced by the weight drop apparatus through the Neurological Severity Score (NSS). The NSS is a comprehensive assessment with a set of clinical criteria, established in 2009 by Flierl et al. (22) and encompasses ten distinct clinical tasks that assesses motor, cognitive, and neurobehavioural performance (Table 1). For each of the ten tasks, a score of one point is awarded for the inability to perform the task. A maximal NSS score of 10 indicates an inability to fulfill all tasks, reflecting severe neurological dysfunction, while a score of zero denotes unimpaired neurological function. NSS assessments were carried out on both *fat-1* and WT mice at 1 h following each WDI. Following the 3rd WDI, NSS assessments were evaluated specifically at 1, 4, 24, 48, 72, and 168 h.

**Table 1:** Neurological severity score (NSS) tasks as described by Flierl et al. (22).

Description	Score
Exit circle	/1
Monoparesis/Hemiparesis	/1
Straight walk	/1
Startle reflex	/1
Seeking behaviour	/1
Beam balance	/1
Round stick balance	/1
Beam walk: 3 cm	/1
Beam walk: 2 cm	/1
Beam walk: 1 cm	/1

Each test is assigned a score of either zero (pass) or 1 (fail). A maximal NSS score of 10 signifies an incapacity to complete all tasks, indicating severe neurological dysfunction. In contrast, a score of zero represents uncompromised neurological function.

### 2.5 GFAP and Prussian blue staining for detection of inflammatory cytokines and cerebral microhemorrhages

Prussian blue (PB) staining for cerebral microhemorrhages and the presence of cellular damage by glial fibrillary acidic protein (GFAP) staining were evaluated 168 h after the last WDI. Brain hemispheres were fixed in 4% formaldehyde and processed in a tissue processor overnight for immunohistochemistry staining. The brain tissues were embedded in a paraffin block, and 5 μm sections were sliced along the dorsoventral plane using a Leica RM2235 microtome. The first 50 μm of brain sections were discarded to reach a point of visibility. Two brain slices each were used to analyze PB (55–60 μm) and GFAP (65–70 μm) appearance. These free-floating tissue sections were then fixed onto Superfrost Plus slides (Cat#12-550-15, Fisher Scientific, Pittsburgh, PA).

Following embedding and slicing of brain tissue, sections intended for PB staining were deparaffinized and hydrated using distilled water before submersion in a solution comprising equal parts of 20% hydrochloric acid (Cat #A144-500, Fisher Chemical) and 10% potassium hexacyanoferrate (II) trihydrate (Cat #P3289-5G, Sigma-Aldrich Canada Co., Oakville, ON) for a duration of 20 min. The sections were then rinsed in 2 successive changes of water and subsequently counterstained with nuclear fast red (Cat #R5463200-500A, Ricca Chemical Company) for 5 min, followed by rinsing in 3 successive changes of water (15). Sections intended for GFAP staining was processed using the Proteintech IHCeasy GFAP Ready-To-Use IHC Kit (Cat #KHC0002). The staining procedure for slides designated for GFAP followed the protocol provided by Proteintech.

After processing for each respective stain of either PB or GFAP, all sections underwent a sequence of baths in the following sequence: dehydration in 95% isopropanol (Cat #HC5001GAL, Fisherbrand™ HistoPrep™); 2 changes in isopropanol for dehydration; and 3 changes of xylene (Cat #HC7001GAL, Fisherbrand™ HistoPrep™), each for a duration of 2 min. Following, sections were mounted with Cytoseal XYL (Cat #8312-4, Epredia, Kalamazoo, MI) and cover slipped (Cat #12-541-023CA, Fisher Scientific, Pittsburgh, PA).

Two independent observers scored PB and GFAP staining of the entire brain slice using a Nikon Eclipse TS100 microscope at 20X and 40X original magnification, respectively. Scores were averaged based on both independent observers. The average number of PB-positive clusters for each brain section was computed following the method described by Fisher et al. (24). Scoring of GFAP staining was based on the stain density of the brain section, which was adapted from the method by Luijerink et al. (25) to allow for greater discrimination between lightly stained sections across a broader range of scores. Intensity of the stain was scored between 0 and 4, with 0 being the least intense and 4 being the most intense.

### 2.6 Statistical analysis

Statistical analysis was conducted using SAS version 9.4 (SAS Institute, Cary, NC, United States). Initially, a two-way analysis of variance (ANOVA) was conducted. In the absence of a significant interaction effect in the number of mTBI, a one-way ANOVA analysis was conducted on the 1 h NSS, righting reflex, and seeking behaviour measurements. Subsequently, Tukey’s Honest Significant Difference (HSD) post-hoc test was used to determine differences between means across each group. NSS was analyzed by repeated measures analysis, followed by a one-way ANOVA with Tukey’s HSD post-hoc test following the 3rd WDI, to detect differences between each group over the 168 h NSS evaluation period. A two-tailed t-test was conducted to identify differences in PB and GFAP staining in WT and *fat-1* mouse brains. Significance was set at *p* ≤ 0.05 for all analyses.

## 3 Results

### 3.1 Time to first movement – righting reflex and seeking behaviour

**Figure 1:**
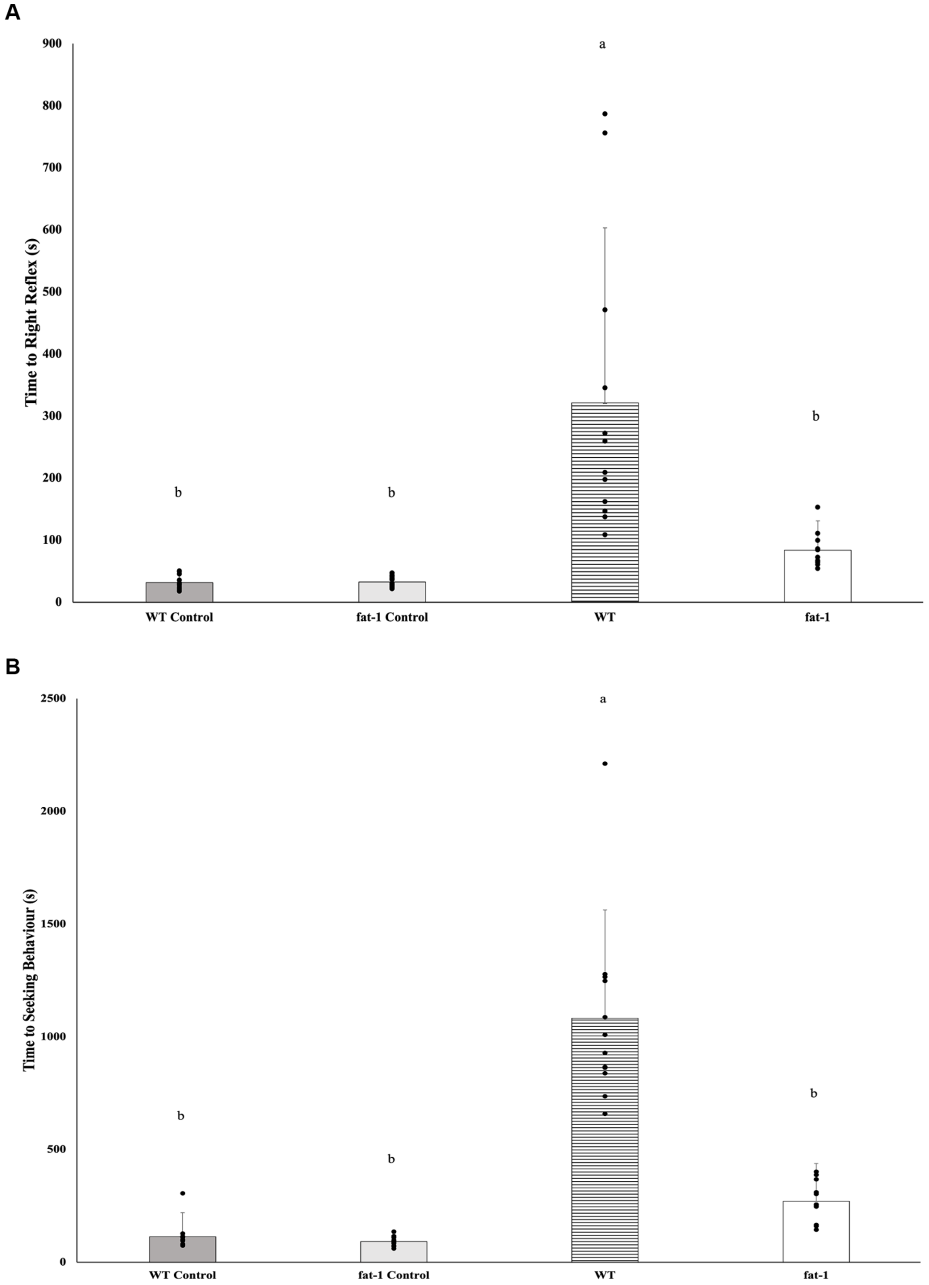
**(A)** Average righting reflex times and **(B)** average time to showing seeking behaviour after three-repeated weight drop injury (WDI) wherein a lower time reflects greater neurological restoration (*n* = 12/group); data analyzed by one-way ANOVA followed by Tukey’s Honest Significant Difference (HSD) post-hoc test. Bars with different letters are significantly different (*p* ≤ 0.05) according to Tukey’s HSD. Data are presented as mean values ± SD.

*Fat-1* mice had significantly faster times to recover the righting reflex (Figure 1A; WT, *n* = 12, 3,211 ± 282 s; *fat-1*, *n* = 12, 84. ± 47 s) and seeking behaviour (Figure 1B; WT, *n* = 12, 1,082 ± 480 s; *fat-1*, *n* = 12, 271 ± 167 s) times following each exposure to the WDI (*p* ≤ 0.05), in comparison to WT mice. *Fat-1* mice were not different in comparison to WT control mice groups (*p* ≥ 0.05).

### 3.2 Neurological severity score

**Figure 2:**
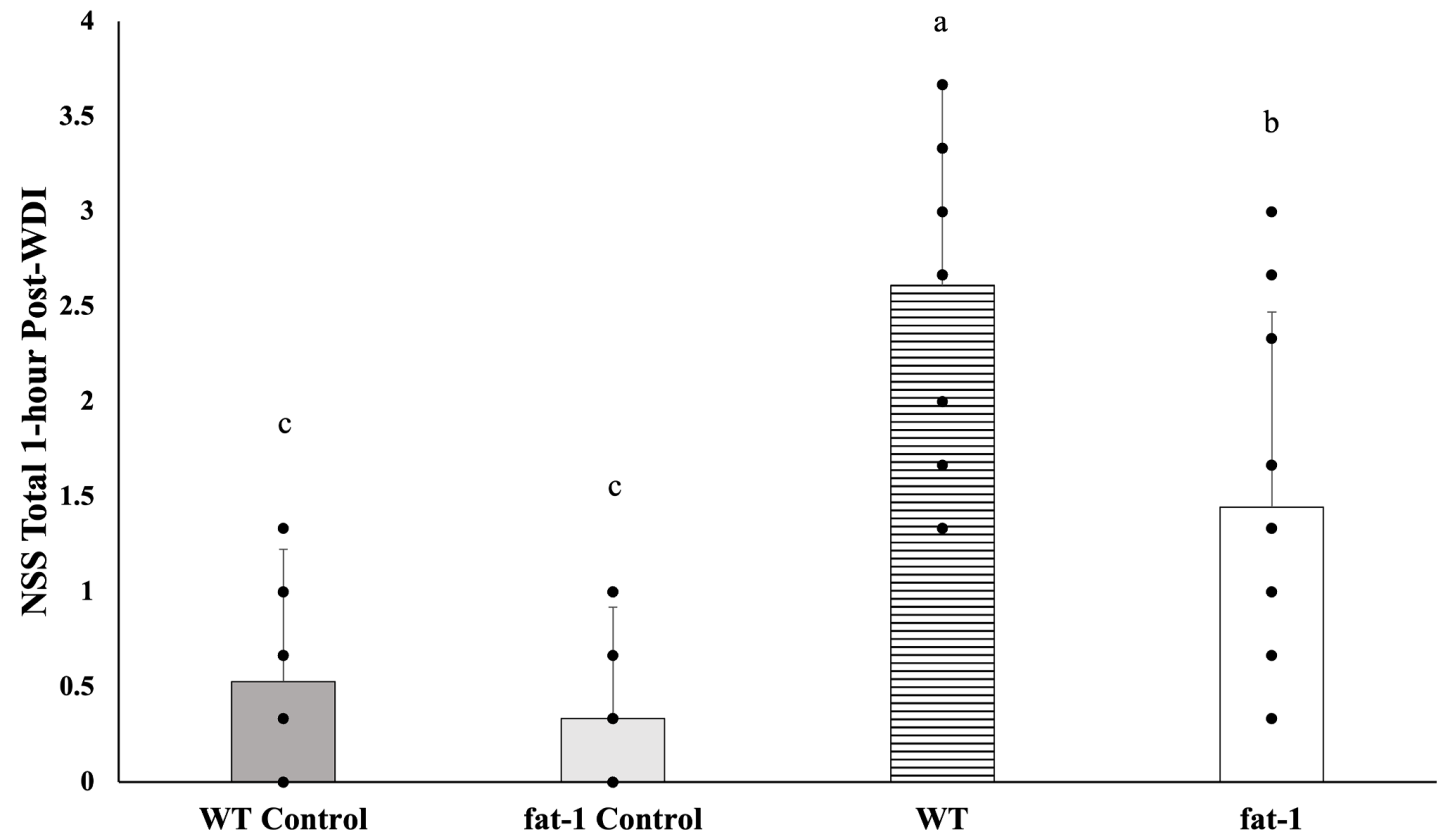
NSS for mice at the 1 h time point following each WDI. *n* = 12 for all groups; wildtype (WT); *fat-*1; WT control and *fat-1* control. Data analyzed by one-way ANOVA with Tukey’s HSD post-hoc test. Bars with different letters are significantly different according to Tukey’s HSD (*p* ≤ 0.05). Data are presented as mean values ± SD.

**Figure 3:**
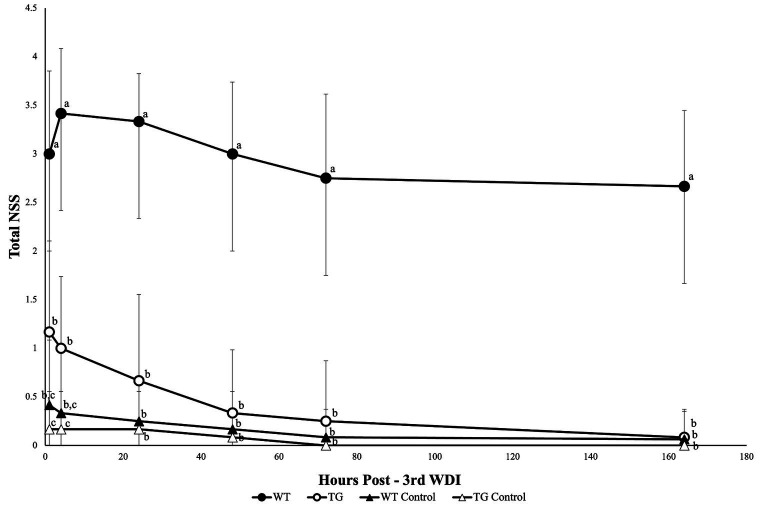
NSS for mice at the 1, 4, 24, 48, 72, and 168 h timepoints following the last WDI. *n* = 12/group; wildtype (WT); *fat-*1; WT control and *fat-1* control. Repeated measures analysis showed a significant difference in NSS (*p* ≤ 0.05) between groups at all timepoints. Subsequently, a one-way ANOVA with Tukey’s HSD post-hoc analysis was conducted at each timepoint to determine differences between groups. Different letters at each time point are significantly different according to Tukey’s HSD (*p* ≤ 0.05). Data are presented as mean values ± SD.

*Fat-1* mice had significantly lower mean NSS scores (*p* ≤ 0.05) at 1 h following each WDI in comparison to WT mice (Figure 2). *Fat-1* mice also had significantly lower mean NSS scores (*p* ≤ 0.05) at all time points post-last WDI; the precise timing of when these differences became significant was at the 48 h time point (Figure 3). Both *fat-1* and WT mice demonstrated continuous enhancements in NSS scores over time. In *fat-1* mice, a progressive decrease in mean NSS score can be seen through the 1 h and 168 h timepoints (mean values ± SD, 1.00 ± 0.74 and 0.08 ± 0.29, respectively). In WT mice, a similar trend can be seen following the 24 h timepoint. However, throughout the evaluation period, the mean NSS score stayed higher for WT mice compared to *fat-1* counterparts. The one-way ANOVA with Tukey HSD post-hoc revealed that the WT group was significantly different (*p* ≤ 0.05) at all time points. Beginning at the 24 h NSS timepoint, *fat-1* mice showed no significant difference with WT control mice (Figure 3).

### 3.3 GFAP and cerebral microhemorrhage

**Figure 4:**
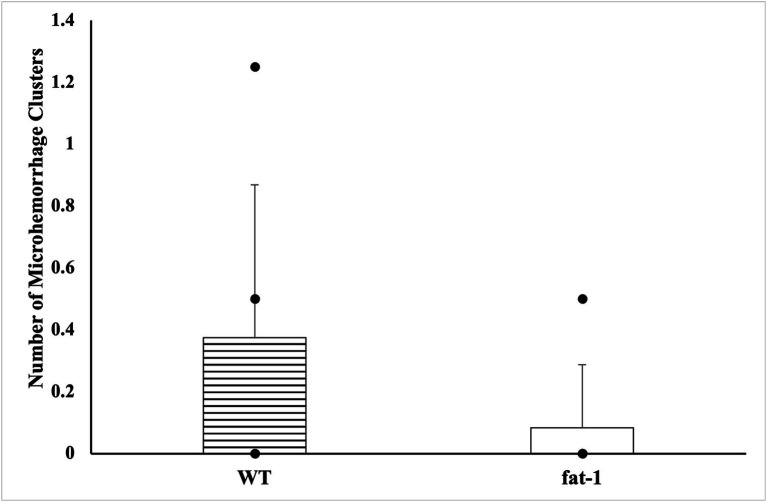
Appearance of cerebral microhemorrhage clusters by Prussian Blue staining for mice at the 168 h timepoint following the last WDI [*n* = 6/group; wildtype (WT); *fat-*1; WT control and *fat-1* control]. WT control and *fat-1* control had no detectable staining and therefore not shown. * denotes a significant difference (*p* ≤ 0.05) by two-tailed *t*-test. Data are presented as mean values ± SD.

**Figure 5:**
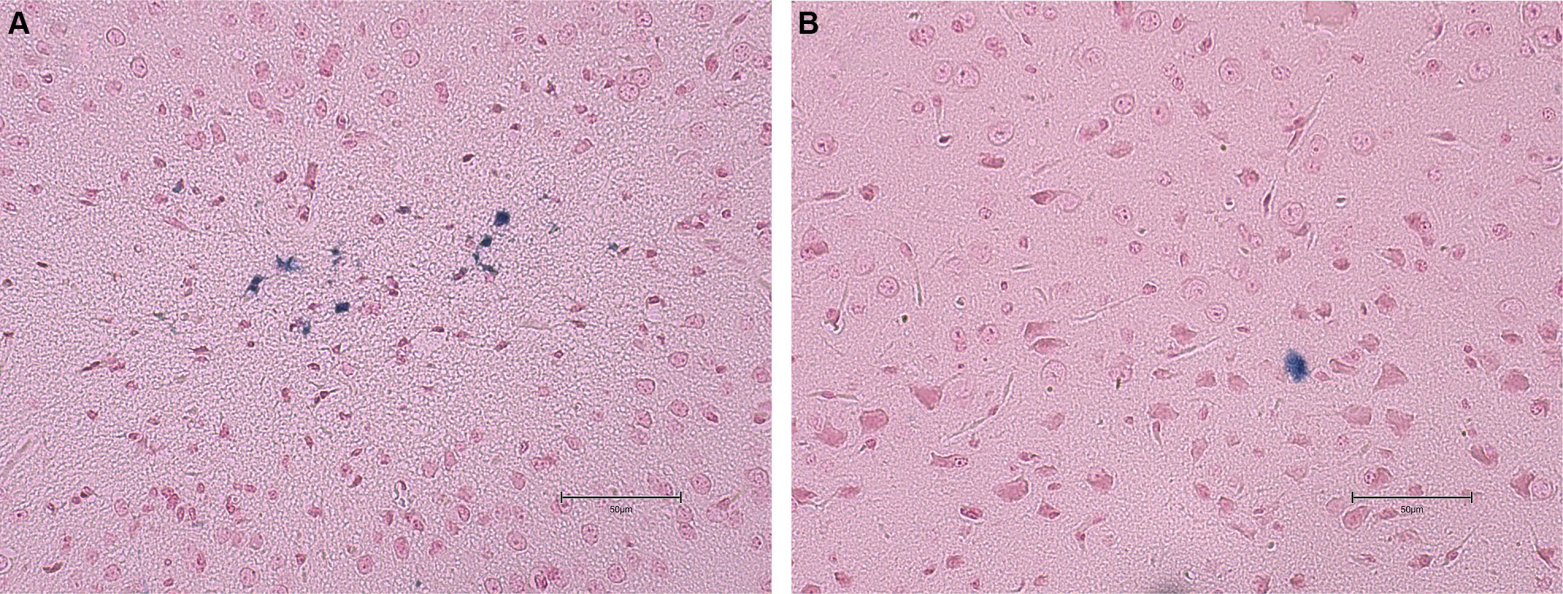
Images of the dorsoventral brain sections of WT and *fat-1* mice stained with Prussian blue 168 h post 3rd WDI. Positive Prussian blue granules are indicative of microhemorrhage. **(A)** WT; **(B)** *fat-1*.

**Figure 6:**
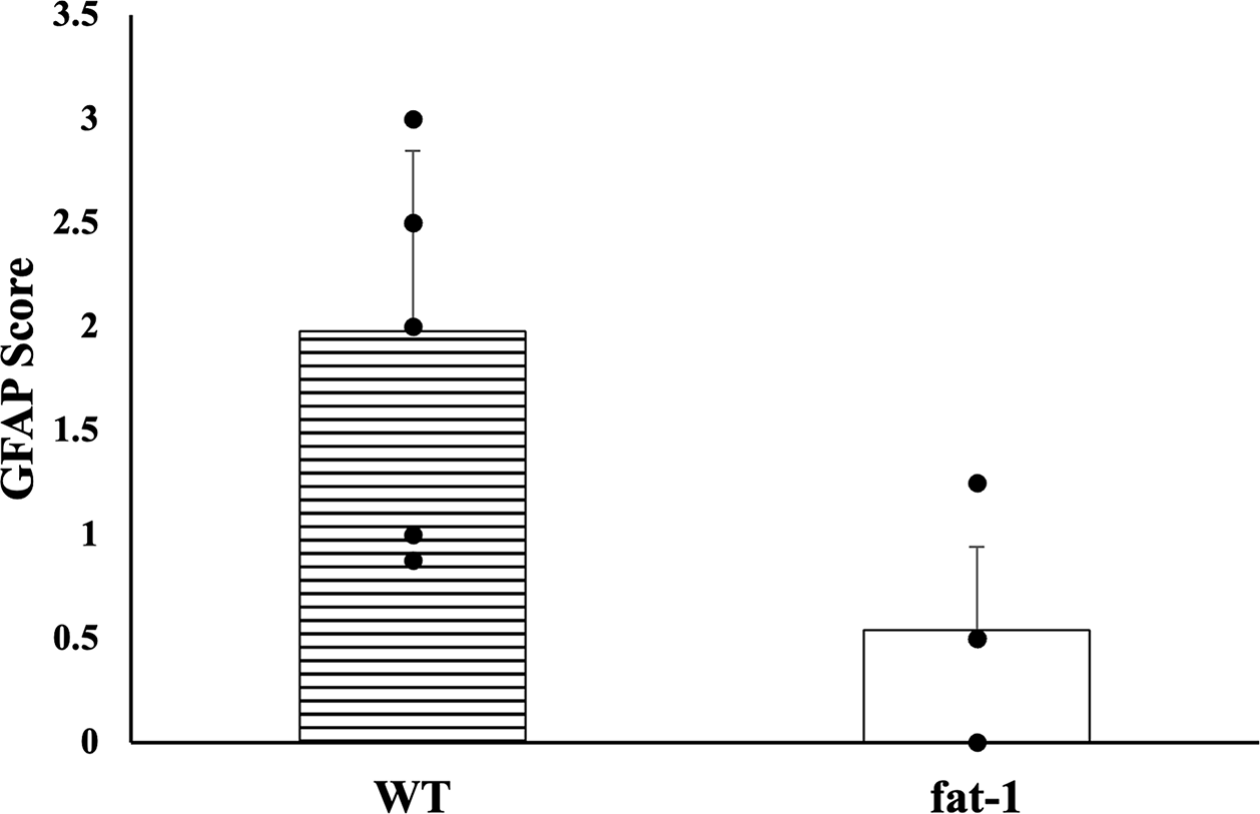
GFAP density for mice at the 168 h timepoint following the last WDI [*n* = 6/group; wildtype (WT); *fat-*1; WT control and *fat-1* control]. WT control and *fat-1* control had no detectable staining and therefore not shown. * denotes a significant difference (*p* ≤ 0.05) by two-tailed *t*-test. Data are presented as mean values ± SD.

**Figure 7:**
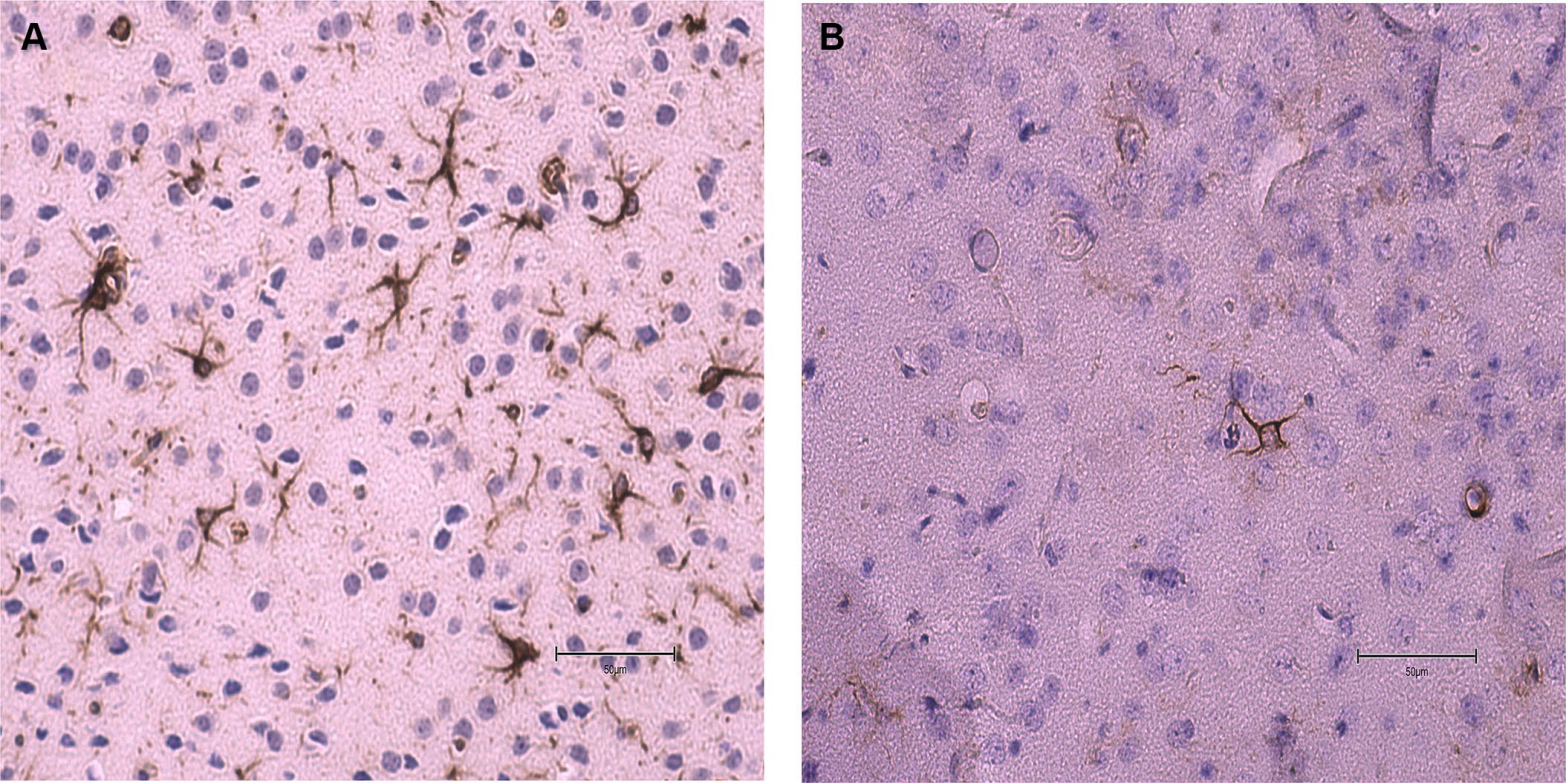
Images of the dorsoventral brain sections of WT and *fat-1* mice stained with GFAP 168 h post 3rd WDI. Dark brown astrocyte appearance is indicative of positive GFAP staining. **(A)** WT; **(B)** *fat-1*.

No statistical differences (*p* ≥ 0.05) were found in the average number of microhemorrhage clusters through PB staining between *fat-1* and WT groups (Figures 4, 5). However, *fat-1* mice had significantly less GFAP staining when compared to WT mice at the 168 h timepoint post-last WDI (Figures 6, 7). Analysis of WT control and *fat-1* control mice found no detectable appearance of staining, resulting in a mean score of zero.

## 4 Discussion

This study showed the potential and causal protective benefit of n-3 PUFA in a model of repeated mTBI in *fat-1* mice. We demonstrated that n-3 PUFA effectively improves neurological recovery, restores cognitive, motor, and behavioural function caused by repetitive WDI. In tandem, lower levels of GFAP, a cellular marker of damage, was observed in *fat-1* mice.

### 4.1 Weight drop injury model

Repeated mTBI in humans has been associated with chronic neurodegeneration (7, 26, 27), whereas isolated incidents may pose little to no harm long-term (28). Following previous work conducted by our lab investigating the effect of n-3 PUFA on a single mTBI (15), the present study sought to determine whether the mitigative effects seen through the previous model would continue with repeated WDI. The model utilized in this study overcomes the intrinsic limitations apparent in other animal models. Studying the pathology resulting from mTBI necessitates a model that integrates forces acting on the brain, aligning with clinical relevance to human injuries (29–31). This includes both linear and rotational acceleration and deceleration forces, leading to diffuse damage rather than focal damage (29, 32, 33). Furthermore, previous examination of biomechanics in head impacts occurring during athletic activities have revealed that the primary factors contributing to mTBI are high-velocity impacts and swift acceleration of the head (29, 32–34). Earlier models including cortical controlled impact (CCI), fluid-percussion injury, and other fixed-head weight drop models are intricate, involve applying a direct load onto the brain, and are expensive methods that do not effectively replicate the required rapid alterations in head acceleration (35–41). These models were initially designed to simulate moderate or severe cases of TBI. Subsequently, studies on mTBI have adjusted these protocols by reducing impact velocity, depth, or duration however, the biochemical limitations of these models do not replicate the outcomes of mTBI in athletic situations (42). Recently, various models incorporating these biomechanics have been developed, diverging from previous traditional models that cause localized damage characteristic of moderate or severe TBI. These models include modified weight drop (MWD), lateral impact (LI), and modified CCI (43). In these newer adaptations of traditional models, subtle adjustments allow for strategies for contacting the head and inducing inertial force, mimicking the conditions associated with mTBI occurring in athletes (43).

In the current study, a mouse model of mTBI was employed, closely replicating the acceleration forces and biomechanical characteristics of head impacts in humans. This was achieved by administering a weight drop impact to the closed cranium of an unrestrained subject, allowing for rapid acceleration of the head and torso, as described by Flierl et al. (22). The current model minimizes the confounding effects on mTBI outcomes associated with extended exposure to anesthetic agents, as it only requires light anesthesia. Since no surgical alterations were required, the process was streamlined, making it both simple and cost-effective. Taking into account these factors and the minor impairments resulting from the impact, it can be contended that this model generates effects comparable to the mechanistic features of human mTBI (21, 35, 44). This, in turn, allows for the investigation and understanding of the neurobiological and neurobehavioural outcomes associated with repetitive mTBI.

### 4.2 *Fat-1* mouse model

In the present study, the utilization of the genetically altered *fat-1* mouse provides the means to investigate the preventive effects of n-3 PUFA in alleviating the pathological sequence that follows a mTBI, all while minimizing the impact of potential confounding factors. Conventionally, dietary supplementation is used to alter the nutrient composition of tissue in nutritional animal studies (21). While it is a broadly acknowledged approach to investigating the effects of nutrients on various physiological processes and pathological conditions, this method introduces various confounding variables. The inherent variations between diets in nutritional studies can lead to inconsistent or conflicting results, especially in studies within the field of lipid nutrition, particularly n-3 PUFA (45). The most challenging aspect of dietary fat studies conducted on an isocaloric basis is ascertaining whether effects are due to the presence of absence of the fatty acid being manipulated. Thus, in the case of studies comparing n-6 and n-3 PUFA, it is not possible to identify the causal relationship between the fatty acid being manipulated and study outcomes. Also, these diets test varying n-3/n-6 ratios by manipulating levels of n-3 PUFA from varying sources of fish oils and plant seed or vegetable oils. Therefore, these oils may also contain traces of additional bioactive compounds (21). Moreover, it is widely acknowledged that PUFA are prone to oxidation, thus antioxidants must be added for storage (46). Hence, numerous variables stemming from diet supplementation have the potential to introduce confounding effects on the profiles of fatty acids and study outcomes.

Our earlier study employed the genetically engineered *fat-1* mouse model to explore how n-3 PUFA can act preventively in reducing the pathological sequence that follows one mTBI (15). In the current study, the *fat-1* mouse model was utilized once again, to reaffirm the causal relationship with repetitive mTBI. The *fat-1* transgenic mouse expresses the *C. elegans fat-1* gene encoding an n-3 fatty acid desaturase which allows for endogenous conversion of n-6 PUFA to n-3 PUFA (47, 48). Tissues of transgenic *fat-1* mice have decreased levels of n-6 PUFA, such as linoleic acid (LA; 18:2n6) and arachidonic acid (AA; 20:4n6), resulting in n-6: n-3 PUFA ratios approaching 1 (49). Previous investigations within our laboratory analyzed the complete brain fatty acid composition in *fat-1* and WT mice, showing significantly higher levels of EPA and DHA in *fat-1* mice (15). Furthermore, transgenic *fat-1* mice exhibit the capability to convert n-6 PUFA to n-3 PUFA from the embryonic stage (49). This eliminates the need for the prolonged and costly feeding periods typically required to alter tissue nutrient profiles in traditional dietary supplementation studies. Thus, this model offers an ideal alternative method to dietary studies to investigate the prophylactic function of n-3 PUFA in mTBI.

### 4.3 Neurological severity score – cognitive, behavioural, and motor function

NSS in this study was consistently lower all post-WDI timepoints, in *fat-1* compared to WT mice. The initial increase in NSS at the earliest timepoint of 1 h, reflected immediate response to the initial injury. Characterization of neurological recovery by NSS at later times post-injury is highly feasibility in the mouse model. In contrast, detecting mTBI or repetitive mTBI in mouse or humans poses a heightened challenge due to its inherent complexity. Common imaging methods such as CT and MRI, for the most part, offer limited assistance in assessing and managing mTBI (50). However, there is promise in more advanced and specialized techniques such as diffusion tensor imaging (51, 52). Selecting suitable neurological assessments is essential in evaluating the impact on both the behavioural and neuroanatomical facets of recovery. These tests must have the capability to identify clinically significant alterations in behaviour that align with underlying biological changes (53). The NSS has undergone prior validation and is recognized as a highly dependable prognostic tool (22, 54). This practical assessment has several properties making it applicable in neurological evaluation as it is easily administered and evaluates various deficits encompassing cognitive, behavioural, and motor aspects (54, 55). Moreover, it exhibits a strong correlation with the severity of brain damage and has been found to align consistently with recovery (22). Thus, different from the rotarod or wire grip test where the focus is typically on one aspect of injury outcome, the NSS provides a comprehensive and reliable method to evaluate the overall neurological status of animals. Its simplicity and accessibility make it stand out as a valuable tool for assessing a wide range of physiological functions.

Previous studies by others investigating the relationship between supplemental n-3 PUFA and TBI also utilized the NSS to study the neuroprotective potential of n-3 PUFA against TBI. The findings revealed that n-3 PUFA can improve the neurological function, as well as learning and memory ability in animals following TBI (56–58). Similarly, in this study, improvements in NSS among *fat-1* mice can be attributed to motor, cognitive, and behavioural function as it comprises tasks that incorporate all these domains. The coordination of movement is facilitated by an intricate network of neural pathways within the brain that travels through various brain structures and the spinal cord, ultimately terminating in skeletal muscle (35, 59). These interconnected regions of cerebral circuitry collaborate to transmit signals through the spinal cord, ultimately regulating motor control through skeletal muscle activity. Motor deficits can result from disturbances caused by brain injury in any or all segments of these neural pathways. Therefore, improvements in motor impairments might be the driving force behind swift reductions in mean NSS noted in *fat-1* mice compared to WT mice following repeated WDI. Future investigations should strive to clarify the precise mechanisms through which n-3 PUFA may alleviate motor deficits. However, recent literature suggests that n-3 PUFA have the ability to enhance information processing and allocated resources more effectively for task demands by improving cortical networking (60).

One significant outcome of mTBI is the impairment of neuronal structures, with a particular focus on axonal damage, leading to potential neuronal apoptosis (61, 62). Such disturbances in the expression of various molecular systems crucial for cognitive function are evident after mTBI. The structural harm to the axon leads to multiple biochemical changes that can impact the uncontrolled release of neurotransmitters following the primary axonal damage, as well as the regulation of genes association with neuronal function (63–65). There are various potential mechanisms to elucidate how elevated levels of circulating n-3 PUFA can counteract the harmful impacts of mTBI (66). One specific mechanism involves the release of the neurotransmitter dopamine, which holds significant roles in learning, memory, and emotion. Fluctuations in dopamine release might be a contributing factor to cognitive and behavioural deficits observed following mTBI. A substantial rise in dopamine levels in the CNS leads to various outcomes, including heightened oxidative stress and the activation of inflammatory signals (63, 67). Conversely, a reduction in dopamine, commonly observed in chronic TBI cases, results in different consequences, such as impairments in memory, attention, and cognitive function. Hence, regardless of fluctuations of dopamine levels increase or decrease dramatically, dopamine can induce notable cellular dysfunction (68). As a result, fluctuations in dopamine levels and associated modifications in dopaminergic systems may exert a profound influence on functional outcomes in cognition and behaviour (68). Previous research indicates that n-3 PUFA can restore dopamine release following brain injury (66). N-3 PUFA protects the brain by minimizing axonal structural damage and neuronal apoptosis while also contributing to the restoration of specific protective mediators, thereby decreasing injury-induced dysfunction (3, 69). Therefore, higher levels of n-3 PUFA observed in *fat-1* mice may have imparted neuroprotective qualities, potentially preserving cognitive and behavioural function. This preservation could explain the overall lower mean NSS scores observed in *fat-1* mice compared to WT mice. However, future experiments exploring the mechanisms underlying neuronal apoptosis and dopamine regulation are required.

### 4.4 Righting reflex and seeking behaviour

In both assessments evaluating the time to display the righting reflex and seeking behaviour, *fat-1* mice subjected to mTBI consistently demonstrated faster times when compared to WT mice. These measurements indicate injury severity (70) thus, the faster times measured suggest n-3 PUFA continues to enhance neurological recovery post-concussion, even after multiple WDI. This is likely attributed to the neuroprotective and neurorestorative qualities associated with n-3 PUFA (71).

In our previous study investigating the mitigative effects of n-3 PUFA on a single mTBI, we measured “time to first movement” as an assessment parameter (15). This metric was employed to evaluate the protective capabilities of n-3 PUFA on neurological recovery. In a prior investigation conducted by Kane et al. (23), the indication of recovery through a righting reflex was assessed as an indicator of neurological restoration. Following the WDI, mice were positioned on their backs in a sanitized cage. Subsequently, the righting reflex was defined as the time it took for the injured mice to naturally revert to a prone position following the injury and/or anesthesia (23). Our investigation adopted a slightly adjusted methodology. This involved incorporating two key metrics: the time taken to reorient themselves rotating from a dorsal to a ventral position (prone position) and the time required to exhibit novelty seeking behaviour. The former metric resembles the “time to first movement” parameter utilized in our previous study following WDI, serving as indicators of neurological recovery. The addition of the seeking behaviour measurement was introduced to assess the restoration of both motor and cognitive functions.

### 4.5 Cerebral microhemorrhage and GFAP

This study employed two approaches to assess brain injury by measuring the prevalence of cerebral microhemorrhage defined by PB and the presence of GFAP. Cerebral microhemorrhages result from bleeding in damaged small cerebral arteries, arterioles, and capillaries, indicating substantial damage in specific anatomical regions and cumulative effects of lesions (72). However, intracranial cerebral microhemorrhages are less common in cases of mTBI (73). Consequently, as mTBI triggers a neuroinflammatory response leading to elevated levels of inflammatory cytokines and axonal injury, measurements of GFAP would be more suggestive of mTBI (74).

The results from the immunohistochemical analysis in this study revealed that, at the 168 h timepoint after the last WDI, there were no significant differences in the prevalence of cerebral microhemorrhages between *fat-1* mice and WT mice. This aligns with prior research suggesting that cerebral microhemorrhages are not a prominent feature of mTBI and do not serve as a significant predictor for cognitive outcomes and post-injury symptoms (75). Additionally, although cerebral microhemorrhages may be present in all severities of TBI, a study by Griffin et al. in 2019 found that microhemorrhages were detected in only 27% of individuals with mTBI, 47% with moderate TBI, and 58% with severe TBI (76). Cerebral microhemorrhages provide a broad overview of injury however, markers of inflammation can elucidate the underlying biological alterations that take place following mTBI.

In this study, the density and intensity of GFAP was significantly lower in *fat-1* mice in comparison to their WT counterparts. GFAP, a cytoskeletal protein specific to astrocytes (77, 78), is a well-established, indicator of glial damage in various traumatic neurological disorders (79–82). Previous studies have consistently documented significant elevated serum levels of GFAP, a well-established marker of glial damage, following TBI. These findings show apparent associations with the severity of injury and subsequent outcomes thus, marking it as a possible diagnostic biomarker (81, 83). While GFAP serves as an indicator of injury in varying severities of TBI, previous studies also revealed that GFAP levels can be identified in serum within 1 hour of injury, reaching their peak at 20 h post-injury, gradually decrease over the subsequent 72 h, and be detectible at 7 days (77); thus, could attribute to lower concentrations of GFAP observed.

Exploring the efficacy of n-3 PUFA in guarding against cerebral microhemorrhages may be more pertinent in the context of moderate to severe TBI, rather than mTBI, as examined in this study. Nevertheless, investigating the hallmark features of mTBI, such as the presence of inflammatory cytokines, provides important specific insights into the injury-specific mechanisms inherent in mTBI.

## 5 Limitations and future directions

This study has provided important insights into the utility of n-3 PUFA in repetitive mTBI. Nevertheless, the current study has several limitations. First, the model used in this study is subject to some variability in impact site than models using a fixed-head model. This may result in variability in outcomes; however, this compromise allows freedom of head movement and rapid acceleration that more closely reflects most human mTBI, and therefore gives more clinically relevant outcomes. Second, another limitation of the study is that only male mice were used and thus sex differences were not examined and future research including females are needed. Lastly, immunohistochemistry was conducted at 168 h post-last WDI. It would be valuable for future studies to sample inflammatory cytokines at earlier timepoints.

## 6 Conclusion

Given the high prevalence of repetitive mTBI and its significant morbidity potential, it is important to address improved treatment, amelioration, or even prevention of the cumulative effects of repetitive mTBI (44, 84). This study was the first to assess the mitigative effects of n-3 PUFA on repetitive mTBI through a genetic approach, utilizing the *fat-1* mouse model. The results from this study demonstrate that n-3 PUFA have the potential to mitigate the motor, neurological, cognitive, and behavioural impairments arising from repetitive concussive injury over 1 week. This work establishes the preventative and protective role of n-3 PUFA in support of brain health. This work has direct clinical relevance and supports the need for the inclusion of n-3 PUFA in the diet as a protective measure for the brain, whether in the context of sports injuries or daily living. However, additional investigation to elucidate the molecular mechanisms governing the action of n-3 PUFA and exploring potential sex-based differences in the utilization of n-3 PUFA as part of protective measures against mTBI is needed.

## Data availability statement

The raw data supporting the conclusions of this article will be made available by the authors, without undue reservation.

## Ethics statement

The animal study was approved by Animal Care and Use Committee of the University of Guelph. The study was conducted in accordance with the local legislation and institutional requirements.

## Author contributions

JL: Data curation, Formal analysis, Investigation, Visualization, Writing – original draft, Writing – review & editing. CL: Writing – review & editing. J-DL: Writing – review & editing, Methodology. LH: Writing – review & editing, Methodology, Project administration, Supervision. MM: Writing – review & editing, Funding acquisition. JK: Writing – review & editing, Conceptualization, Data curation, Formal analysis, Funding acquisition, Investigation, Methodology, Project administration, Resources, Software, Supervision, Validation, Visualization. LR: Writing – review & editing, Conceptualization, Funding acquisition, Methodology, Project administration, Resources, Supervision. DM: Conceptualization, Writing – review & editing, Funding acquisition, Methodology, Project administration, Resources, Supervision.
